# Predicting plant growth response under fluctuating temperature by carbon balance modelling

**DOI:** 10.1038/s42003-022-03100-w

**Published:** 2022-02-24

**Authors:** Charlotte Seydel, Julia Biener, Vladimir Brodsky, Svenja Eberlein, Thomas Nägele

**Affiliations:** 1 Ludwig-Maximilians-Universität München, Faculty of Biology, Plant Development, 82152 Planegg-Martinsried, Germany; 2 Ludwig-Maximilians-Universität München, Faculty of Biology, Plant Evolutionary Cell Biology, 82152 Planegg-Martinsried, Germany

**Keywords:** Dynamic networks, Plant sciences

## Abstract

Quantification of system dynamics is a central aim of mathematical modelling in biology. Defining experimentally supported functional relationships between molecular entities by mathematical terms enables the application of computational routines to simulate and analyse the underlying molecular system. In many fields of natural sciences and engineering, trigonometric functions are applied to describe oscillatory processes. As biochemical oscillations occur in many aspects of biochemistry and biophysics, Fourier analysis of metabolic functions promises to quantify, describe and analyse metabolism and its reaction towards environmental fluctuations. Here, Fourier polynomials were developed from experimental time-series data and combined with block diagram simulation of plant metabolism to study heat shock response of photosynthetic CO_2_ assimilation and carbohydrate metabolism in *Arabidopsis thaliana*. Simulations predicted a stabilising effect of reduced sucrose biosynthesis capacity and increased capacity of starch biosynthesis on carbon assimilation under transient heat stress. Model predictions were experimentally validated by quantifying plant growth under such stress conditions. In conclusion, this suggests that Fourier polynomials represent a predictive mathematical approach to study dynamic plant-environment interactions.

## Abstract (web summary)

Fourier polynomials can be used to model the stress response of carbon balancing in plants. This work suggests that Fourier polynomials could be used to predict plant responses in varied environmental conditions.

## Introduction

Capturing dynamics in biological systems by mathematical terms is the general aim of biomathematical modeling. Differential equations represent an adequate strategy to describe dynamics over space and time. Ordinary differential equations (ODEs) and partial differential equations (PDEs) have been successfully applied to reveal biological system dynamics and to develop predictive models of growth rates, transcription, translation, or metabolic processes^1^. In a metabolic context, ODE models are frequently applied to simulate enzyme kinetic reactions and, by this, to explain dynamics of observed metabolite concentrations. Kinetic models, based on ODEs, have frequently been applied in a broad field of biological research, e.g., in the context of metabolic engineering of microbial systems and strain design^2^, disease research^3^, and plant metabolism^4–6^. In contrast to the high diversity of application fields, the principle of ODE kinetic modeling remains conserved: based on genome sequence information or biochemical evidence from literature a biochemical reaction network is established, substrate and product concentrations are quantified, and enzyme kinetics are applied to calculate reaction rates within a metabolic system. Enzyme kinetic parameters, e.g., velocity under substrate saturation (*V*_max_) or substrate affinity (*K*_M_) of Michaelis–Menten equations, are experimentally determined and used for computationally assisted parameter estimation. A well-defined and experimentally validated kinetic ODE model enables computational simulation and prediction of complex system behavior. A clear limitation of such an approach, however, is the requirement of kinetic parameters which are (frequently) difficult and/or expensive to quantify. Initiatives like KiMoSys, a public repository of published experimental data, summarize and concentrate data on metabolites, protein abundance, and fluxes providing a solid database for model construction and initial development^7^. Yet, under highly dynamic conditions, e.g., in a fluctuating environment, it still remains a challenge to resolve system dynamics on an enzyme kinetic level. This is due to the high dynamics of metabolites, transcripts, protein levels, and enzyme activities^8,9^. Although being laborious, the development and optimization of ODE kinetic models provide an important and informative mathematical method to study biochemical system behavior. Simultaneously, however, diverse problems might occur with solving and applying such models due to uncertainties about parameters, model structure, kinetic rate laws or parameter sensitivities^10,11^. Depending on the research question focused on by a study, an explicit knowledge about enzymatic activities and their dynamics might not be essential to derive a mathematical description of metabolite dynamics. For example, metabolic fluxes might be estimated by tracing labeled atoms or molecules in a metabolic pathway system^12^. While only very limited information about single enzyme activities or kinetics can be derived from flux estimations, they still provide comprehensive insights into metabolic states and pathway activities, also on a large scale^13^. Beyond, algorithms and user interfaces have been developed which enable the combination of flux data with relative metabolite levels^14^.

For estimating metabolic functions, i.e., the sum of synthesizing and degrading/consuming reactions of metabolite pools, under dynamic environmental conditions we have previously suggested a method for implicit estimation of metabolic functions^15^. Similar to flux analysis, dynamics of metabolite concentrations in time-series experiments were used in this approach to derive a time-continuous mathematical function to identify regulatory cascades in metabolic pathways. This approach made use of spline interpolations which were composed of cubic polynomials which were fitted to adjacent pairs of data points in a time-series data set. While such an approach is suitable for accurate data fitting, underlying mathematical functions are frequently not related to biological function and, thus, are less predictive than enzyme kinetic models. In the present study, we developed a mathematical model based on Fourier polynomials to simulate and analyze dynamics of photosynthesis and carbohydrate metabolism under transient heat exposure by function superposition. Model simulations indicated a significant impact of sucrose and starch biosynthesis on the stabilization of carbon assimilation and growth under elevated temperatures.

## Results

### A block diagram model based on Fourier polynomials for carbon balancing of plant metabolism

**Fig. 1:**
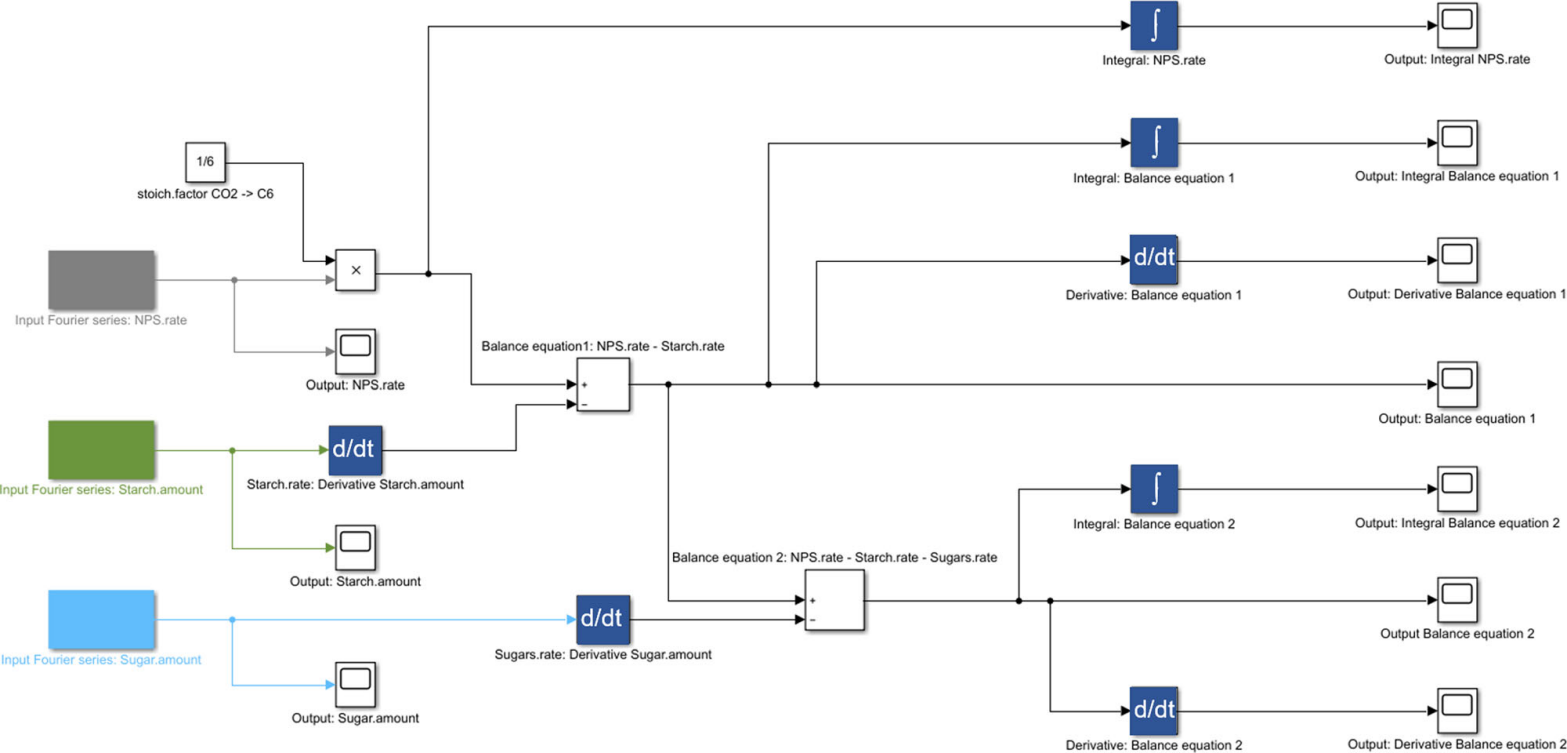
Block diagram applied for Fourier polynomial balance modeling. Input functions are marked in gray (NPS), green (starch amount), and light blue (sugar amount) colored blocks (left side). Arrows indicate the direction of flux and connect input blocks via multiplication (“x”) summation (“+/-“), differentiation (“d/d*t*”), and integration (“∫”) with output blocks.

**Fig. 2:**
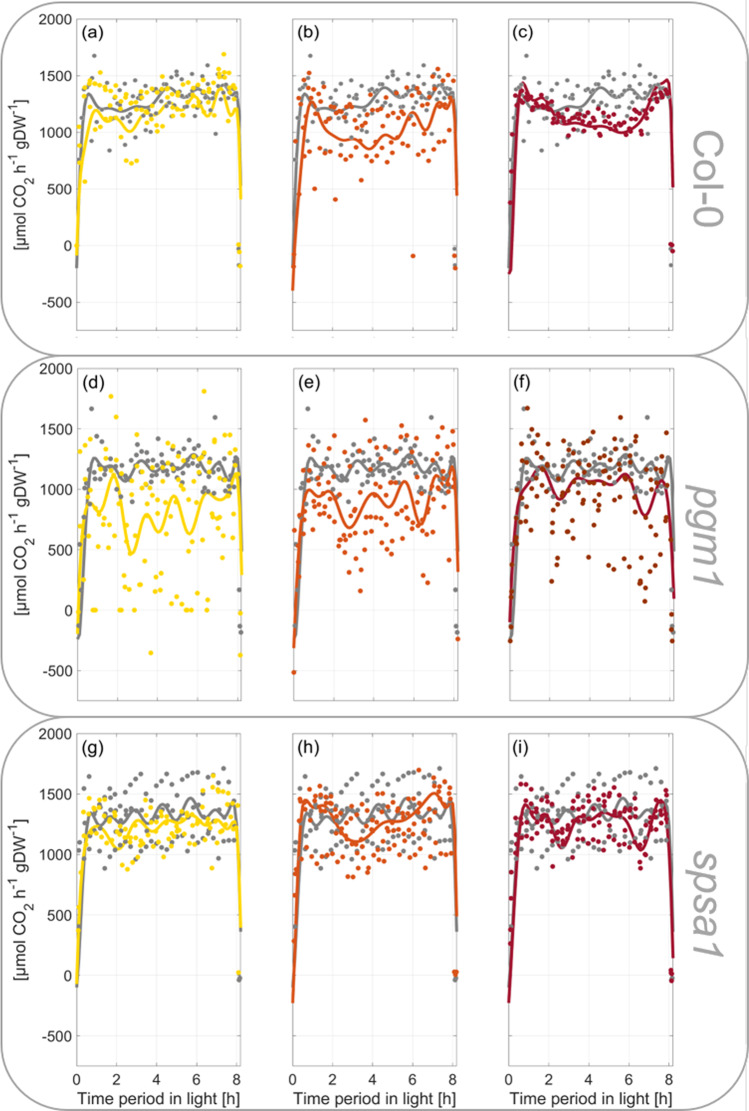
Rates of net CO_2_ uptake during short-day transient heat exposure. Scattered dots represent experimental data (*n* = 3), lines represent Fourier series fits. **a**–**c** Col-0, **d**–**f** *pgm1*, **g**–**i** *spsa1*. Gray lines: 22 °C experiment; yellow lines: 32 °C experiment; orange lines: 36 °C experiment; red lines: 40 °C experiment. The temperature was set to 22 °C between 0–2 h and 6–8 h. The temperature was transiently increased between 2 and 6 h. Temperature curves recorded during the experiments are illustrated in Supplementary Fig. S3a. A summary of Fourier polynomial coefficients is provided in the supplements together with NPS data (Supplementary Data 1 and Supplementary Data 2).

A block diagram model of the central carbohydrate metabolism of plants was developed to integrate experimental data on net photosynthesis (NPS), starch, and sugar metabolism (Fig. 1). The net carbon input block, i.e., NPS block, represented a Fourier polynomial describing NPS dynamics depending on genotypes and environments (see Fig. 2, solid lines). This input flux was multiplied by the stoichiometric factor 1/6 to enable quantitative summation with starch and sugar fluxes (unit: µmol C6 h^−1^ gDW^−1^). Carbon balance Eq. (1) (BE_1_) comprised the summation of NPS rates and negative starch rates, balance Eq. (2) (BE_2_) additionally comprised summation of negative sugar rates (Fig. 1). As a result, BE_1_ revealed the net carbon flux (in C6 equivalents per hour and gram dry weight) which was left from photosynthetically assimilated CO_2_ after starch synthesis, e.g., for sugar biosynthesis or biomass production. Further, BE_2_ revealed residual net carbon flux after additional sugar biosynthesis.

Rates of net starch and sugar biosynthesis were determined by differentiating Fourier polynomials of starch and sugar dynamics with respect to time. Hence, the Fourier polynomial balance models comprised three input functions, FP_input_ (Eqs. (1–3)), and two balance equations, BE_1,2_ (Eqs. (4) and (5)).

1 $$F{P}_{input,NPS}={a}_{0,NPS}+\mathop{\sum }\limits_{k=1}^{n}[{a}_{k,NPS}\,\cos (k{\omega }_{NPS}t)+{b}_{k,NPS}\,\sin (k{\omega }_{NPS}t)]$$

2 $$F{P}_{input,Starch}={a}_{0,Starch}+\mathop{\sum }\limits_{k=1}^{n}[{a}_{k,Starch}\,\cos (k{\omega }_{Starch}t)+{b}_{k,Starch}\,\sin (k{\omega }_{Starch}t)]$$

3 $$F{P}_{input,Sugars}={a}_{0,Sugars}+\mathop{\sum }\limits_{k=1}^{n}[{a}_{k,Sugars}\,\cos (k{\omega }_{Sugars}t)+{b}_{k,Sugars}\,\sin (k{\omega }_{Sugars}t)]$$

4 $$B{E}_{1}=\left(\frac{1}{6}\right)\cdot F{P}_{input,NPS}-\frac{d(F{P}_{input,Starch})}{dt}$$

5 $$B{E}_{2}=B{E}_{1}-\frac{d(F{P}_{input,Sugars})}{dt}$$

Here, *a*_*k*_ and *b*_*k*_ represent the Fourier coefficients for NPS, starch, and sugar equations. ω is the fundamental frequency of the signal (ω = 2π/*T*, where *T* is the period). This Fourier polynomial-based balance equation model was applied to simulate dynamics of carbohydrate metabolism in plants of *Arabidopsis thaliana*, accession Columbia-0, under transient heat exposure. In addition, NPS and carbohydrate dynamics were recorded and simulated in a starch-deficient mutant *pgm1* and a mutant with a deficiency in sucrose biosynthesis capacity, *spsa1*. Coefficients of Fourier polynomials are provided in the supplements (Supplementary Data 1).

### Fourier polynomials reflect dynamics of net CO_2_ assimilation rates

During the first 30–45 min of the light period, rates of net CO_2_ assimilation increased steeply in all genotypes and reached a first plateau at ~1250 µmol CO_2_ h^−1^ gDW^−1^ which was stable during the first half of the light period before it slightly increased until the end of the day at 22 °C (Fig. 2, gray-colored lines; data provided in Supplementary Data 2). No significant difference was observed between genotypes, yet *spsa1* had slightly higher assimilation rates compared to Col-0 while rates of *pgm1* were slightly lower (Fig. 2a, d, g). Temperature increase from 22 to 32 °C resulted in a drop in assimilation rates during the first hour of the treatment before the rates stabilized again and reached similar values than in the control (22 °C) experiment (Fig. 2a, d, g). At 32 °C, starch-deficient *pgm1* plants were most susceptible, and mean values differed most from 22 °C rates (Fig. 2d). During the last 2 hours of the light period in which temperature was decreased to 22 °C, all genotypes increased assimilation rates to control rates again. A similar scenario was observed within the 36 °C experiment for *pgm1* and *spsa1* while Col-0 had significantly decreased assimilation rates during the last 2 h of temperature treatment compared to the control experiment (Fig. 2b). In *pgm1*, assimilation rates dropped significantly during the first 30 min of the recovery phase, i.e., between 6 h and 6.5 h, when the temperature was decreased from 36 °C to 22 °C (Fig. 2e). Such a significant recovery drop was also observed for both *pgm1* and *spsa1* mutants in the 40 °C experiment, but not for Col-0 which showed again significantly decreased CO_2_ assimilation rates between the last 2 h of the temperature treatment, i.e., between 4 and 6 h of the light period (Fig. 2c, f, i). Experimentally determined mean values of CO_2_ assimilation rates and, by this, all described effects were covered by Fourier polynomials with *R*^2^ > 0.94 (exception: *pgm1*, 32 °C, *R*^2^ = 0.8177). In contrast to significant genotype-effects in net CO_2_ assimilation under temperature fluctuation, transpiration rates were similar in Col-0, *spsa1* and *pgm1* under each tested condition and no significant genotype effect was detected (Supplementary Fig. S1). Transpiration rates increased during temperature treatment (2 h→6 h) and decreased again with temperature during the last 2 h of the light phase (6 h→8 h). Peak values of transpiration rates at 40 °C were about ~threefold higher than at 22 °C (Supplementary Fig. S1a, d).

**Fig. 3:**
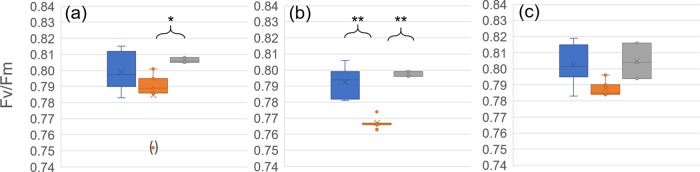
Maximum photochemical quantum yield of PSII (Fv/Fm) under transient heat. Fv/Fm under 22 °C during the first 2 h of the light phase (blue), during transient exposure to 40 °C (orange) and after 2 h of recovery at 22 °C (gray). **a** Col-0, **b** *pgm1*, **c** *spsa1*. Box-and-whisker plots: center line, median; box limits, upper and lower quartiles; whiskers, 1.5× interquartile range; points, outliers. Asterisks indicate significant differences (ANOVA; *P < 0.05; **P < 0.01). *n* = 3–6. Experimental data are provided in Supplementary Data 3.

**Fig. 4:**
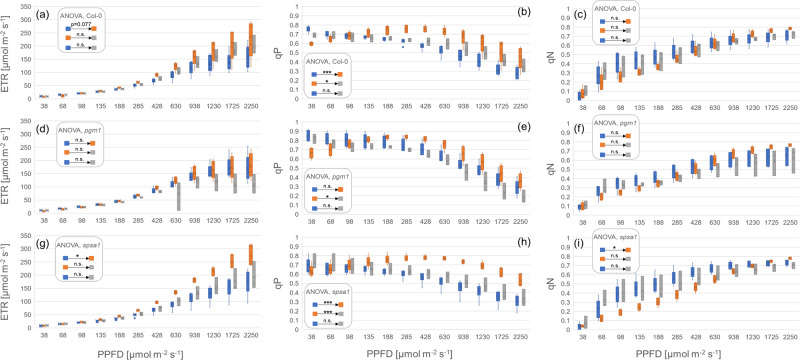
Electron transport rates and quenching parameters under transient heat. Electron transport rates (ETR), photochemical (qP), and non-photochemical (qN) quenching were recorded within a rapid light curve (RLC) protocol. Blue: at 22 °C during the first 2 h of the light phase. Left panel: ETR; middle panel: qP; right panel: qN. Orange: during exposure to 40 °C. Gray: after 2 h recovery at 22 °C. **a**–**c** Col-0, (**d**–**f**) *pgm1*, (**g**–**i**) *spsa1*. Box-and-whisker plots: center line, median; box limits, upper and lower quartiles; whiskers, 1.5× interquartile range; points, outliers. *n* = 3–6. Significances, revealed by ANOVA, are summarized in boxes; n.s.: not significantly different (*P* > 0.05); *P < 0.05; ***P < 0.001. Experimental data are provided in Supplementary Data 3.

To test whether differential efficiency of photosystems could reflect observed differences in CO_2_ assimilation under transient heat, maximum quantum yield (Fv/Fm), electron transport rates (ETR), photochemical (qP), and non-photochemical quenching parameters (qN) were determined by pulse-amplitude modulation before and after transient exposure to 40 °C (Figs. 3 and 4; data provided in Supplementary Data 3). While Col-0 was affected significantly in Fv/Fm only during recovery from transient 40 °C treatment at 22 °C (Fig. 3a), Fv/Fm of *pgm1* dropped significantly during 40 °C treatment and showed a significant increase during recovery (Fig. 3b). In *spsa1*, no significant effect was observed for Fv/Fm (Fig. 3c). In contrast, *spsa1* was most significantly affected in electron transport rates (ETR), photochemical (qP), and non-photochemical quenching (qN) parameters recorded within rapid light curves (RLCs; Fig. 4g–i). At 40 °C, ETR and qP were significantly higher than at 22 °C, also under high PPFD, i.e., >1000 µmol photons m^−2^ s^−1^ (Fig. 4g, h). A similar trend was also observed in Col-0 where qP was also found to increase significantly under transient exposure to 40 °C (Fig. 4b). In *pgm1*, photosystems were found to be least significantly affected (Fig. 4d–f). Here, only qP showed a significant drop when plants were transferred from 40 to 22 °C (Fig. 4e).

### Transient heat exposure significantly affects dynamics of starch and soluble carbohydrates

**Fig. 5:**
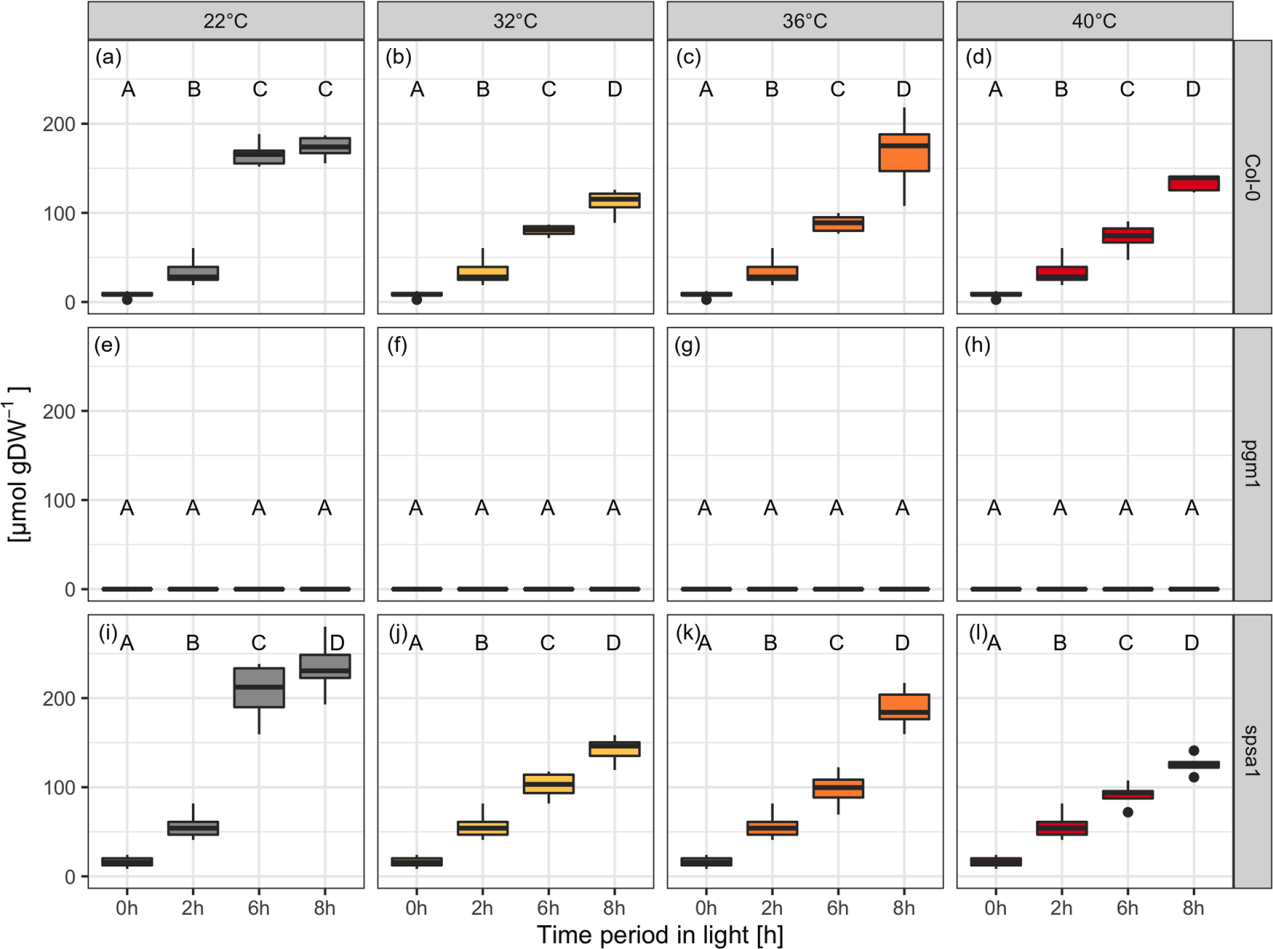
Starch amounts during short-day transient heat exposure in glucose equivalents. **a**–**d** Col-0 (*n* ≥ 5); **e**–**h** *pgm1* (*n* ≥ 3); **i**–**l** *spsa1* (*n* ≥ 5). Gray: 22 °C experiment; yellow: 32 °C experiment; orange: 36 °C experiment; red: 40 °C experiment. The temperature was set to 22 °C between 0–2 h and 6–8 h. The temperature was transiently increased between 2 and 6 h. Box-and-whisker plots: center line, median; box limits, upper and lower quartiles; whiskers, 1.5× interquartile range; points, outliers. Capital letters indicate groups of significance within genotype and condition (ANOVA, *P* < 0.05). Experimental data are provided in Supplementary Data 4.

The exposure to transient heat lead to a significant change in starch dynamics in Col-0 and *spsa1* (Fig. 5; all metabolite data are provided in Supplementary Data 4). Starch amount in *pgm1* was below the detection limit of the applied photometric detection method (Fig. 5e–h). Starch concentration dropped significantly after transient heat exposure (6 h) in comparison to control conditions in Col-0 and *spsa1* (ANOVA, *P* < 0.001). This drop did not change significantly among the different temperatures. In the recovery phase after the heat shock (8 h), plants increased their starch content depending on the temperature they were subjected to. Plants of both genotypes treated with 32 °C increased their starch concentration by ~40% between 6 and 8 h (Fig. 5b, j), whereas plants treated with 36 °C increased it by over 90% (Fig. 5c, k). Col-0 subjected to the 36 °C transient heat exposure was even able to reach the level of the control plants at the 8-h time point.

**Fig. 6:**
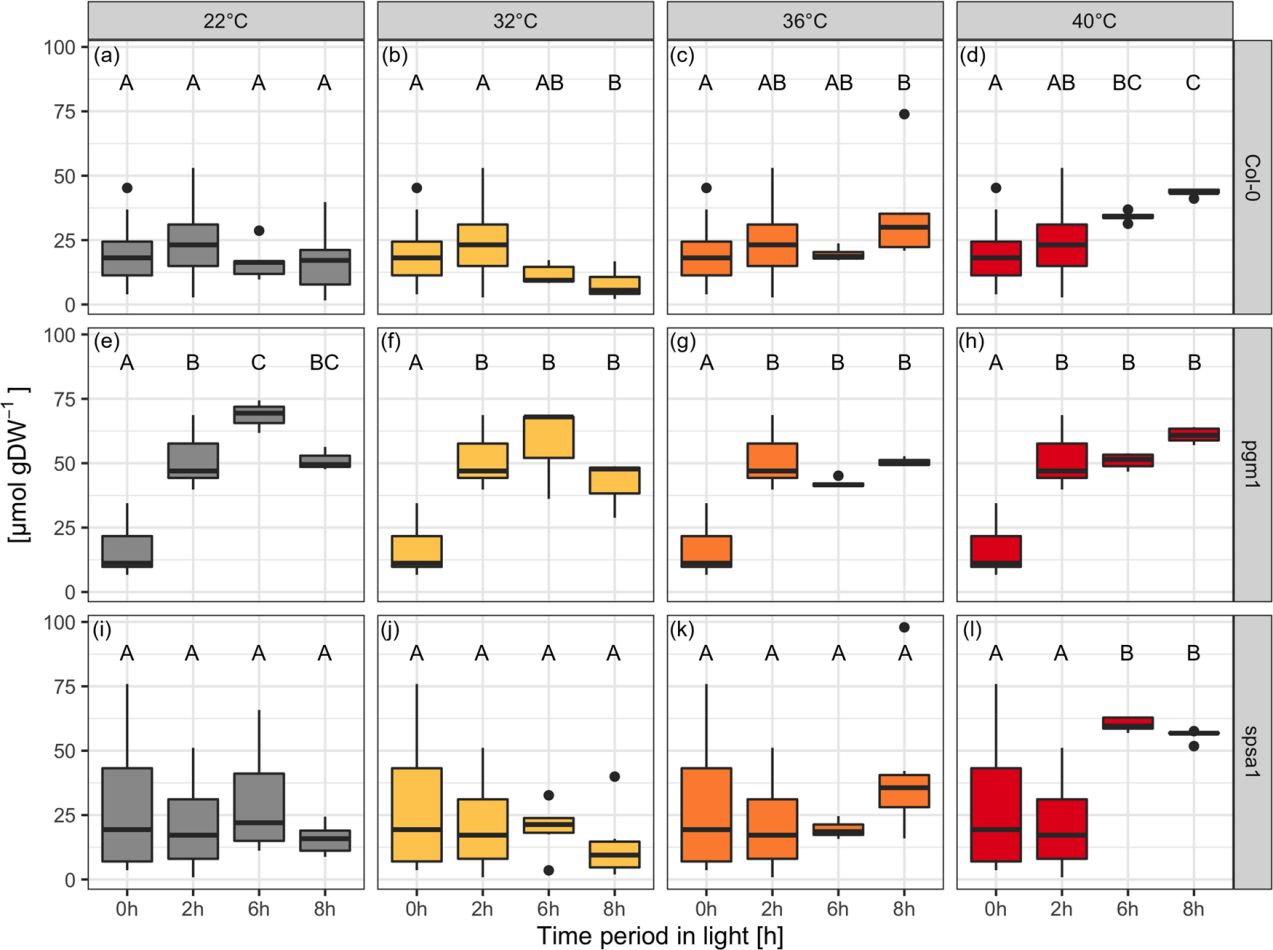
Sucrose concentrations during short-day transient heat exposure. **a**–**d** Col-0 (*n* ≥ 5); **e**–**h** *pgm1* (*n* ≥ 3); **i**–**l** *spsa1* (*n* ≥ 5). Gray: 22 °C experiment; yellow: 32 °C experiment; orange: 36 °C experiment; red: 40 °C experiment. The temperature was set to 22 °C between 0–2 h and 6–8 h. The temperature was transiently increased between 2 and 6 h. Box-and-whisker plots: center line, median; box limits, upper and lower quartiles; whiskers, 1.5× interquartile range; points, outliers. Capital letters indicate groups of significance within genotype and condition (ANOVA, *P* < 0.05). Experimental data are provided in Supplementary Data 4.

An effect of transient heat exposure on sucrose levels was only detectable for higher temperatures, i.e., within 36 °C and 40 °C experiments (Fig. 6). When subjected to 32 °C of transient heat, no significant change in sucrose levels was detected in all genotypes (Fig. 6b, f, j). In Col-0, only heat exposure of 40 °C resulted in a significant increase in sucrose concentration at the 6 h time point (*P* < 0.001), but no further change was observed after the recovery phase at 8 h (Fig. 6d). Under all conditions, *pgm1* accumulated more sucrose over the course of the light phase compared to Col-0 and *spsa1* (Fig. 6e–h). Nevertheless, heat treatment with 36 and 40 °C reduced the amount of sucrose in the *pgm1* plants almost significantly (*P* = 0.05). In the recovery phase, the sucrose concentration in the heat-treated *pgm1* plants returned to a level comparable to control conditions (Fig. 6e–h). Due to high variance in the sucrose measurements of *spsa1* plants, there was no significant difference between Col-0 and *spsa1* in the control plants and the plants exposed to 32 and 36 °C of transient heat (Fig. 6i–k). Only at 40 °C, *spsa1* showed significantly higher sucrose levels than Col-0 at 40 °C before and after the recovery phase (6 and 8 h, *P* < 0.001) or *spsa1* under control conditions (6 h: *P* < 0.004, 8 h: *P* < 0.01), while exhibiting very low variance (Fig. 6l).

**Fig. 7:**
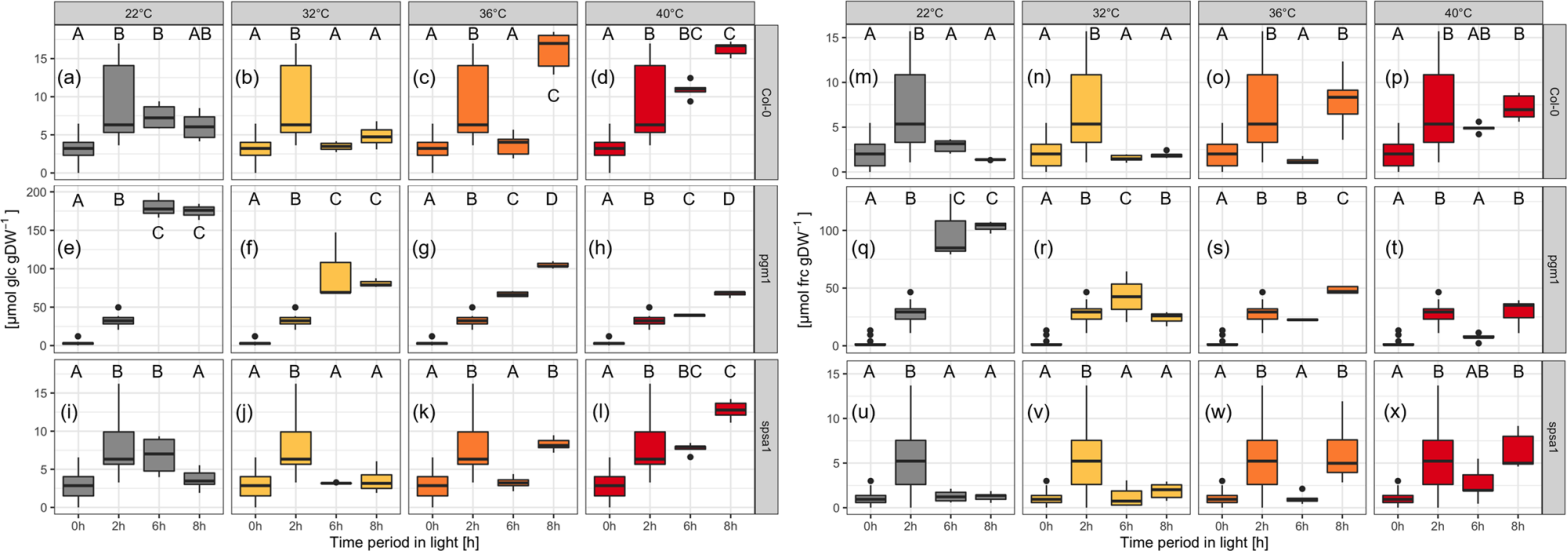
Hexose concentrations during short-day transient heat exposure. Range of the *y* axes differs for *pgm1* due to the high difference in concentration. **a**–**l** Glucose concentrations; **a**–**d** Col-0 (*n* ≥ 5); **e**–**h** *pgm1* (*n* ≥ 3); **i**–**l** *spsa1* (*n* ≥ 5). **m**–**x** Fructose concentrations. **m**–**p** Col-0 (*n* ≥ 5); **q**–**t** *pgm1* (*n* ≥ 3); **u**–**x** *spsa1* (*n* ≥ 5). Gray: 22 °C experiment; yellow: 32 °C experiment; orange: 36 °C experiment; red: 40 °C experiment. The temperature was set to 22 °C between 0–2 h and 6–8 h. The temperature was transiently increased between 2 and 6 h. Box-and-whisker plots: center line, median; box limits, upper and lower quartiles; whiskers, 1.5× interquartile range; points, outliers. Capital letters indicate groups of significance within genotype and condition (ANOVA, *P* < 0.05). Experimental data are provided in Supplementary Data 4.

In *pgm1*, glucose and fructose dynamics differed significantly from Col-0 (Fig. 7). Starting at similar hexose content at the start of the light period, the difference between *pgm1* and Col-0 and *spsa1* increased almost tenfold over the course of the day. Whilst in *pgm1* hexose amount increased steeply under control conditions, reaching a plateau after 6 h, hexose concentrations in Col-0 and *spsa1* peaked after 2 h and subsequently decreased again.

Col-0 and *spsa1* showed a significant drop in glucose levels after heat exposure to 32 and 36 °C (*P* < 0.001, Fig. 7). Within the 32 °C experiment, glucose levels at 8 h after recovery did not differ significantly from control conditions. Within the 36 and 40 °C experiments, however, the recovery phase after heat exposure resulted in a significant increase in glucose concentration compared to control conditions (*P* < 0.001). In addition, in Col-0 glucose levels were increasing above the level of control plants already during heat exposure to 40 °C at 6 h (*P* < 0.001). Fructose dynamics in Col-0 were similar to the glucose dynamics in Col-0 (Fig. 7). In *spsa1*, however, fructose dynamics did not change significantly in response to transient heat exposure. The only differences were observable after the recovery phase at 8 h in 36 and 40 °C. Here, a significantly higher fructose content could be measured compared to 22 °C (*P* < 0.001). In *pgm1*, hexose levels decreased significantly after temperature treatment (6 h, *P* < 0.001), with the lowest values being reached at 40 °C. After recovery (8 h), hexose levels did not change significantly from the 6 h time point in the 32 °C plants. After exposure to 36 and 40 °C, however, hexose levels increased significantly from 6 to 8 h (*P* < 0.001).

### Numerical differentiation and integration of carbon balance equations reveals genotype-dependent system fluctuations due to transient heat exposure

**Fig. 8:**
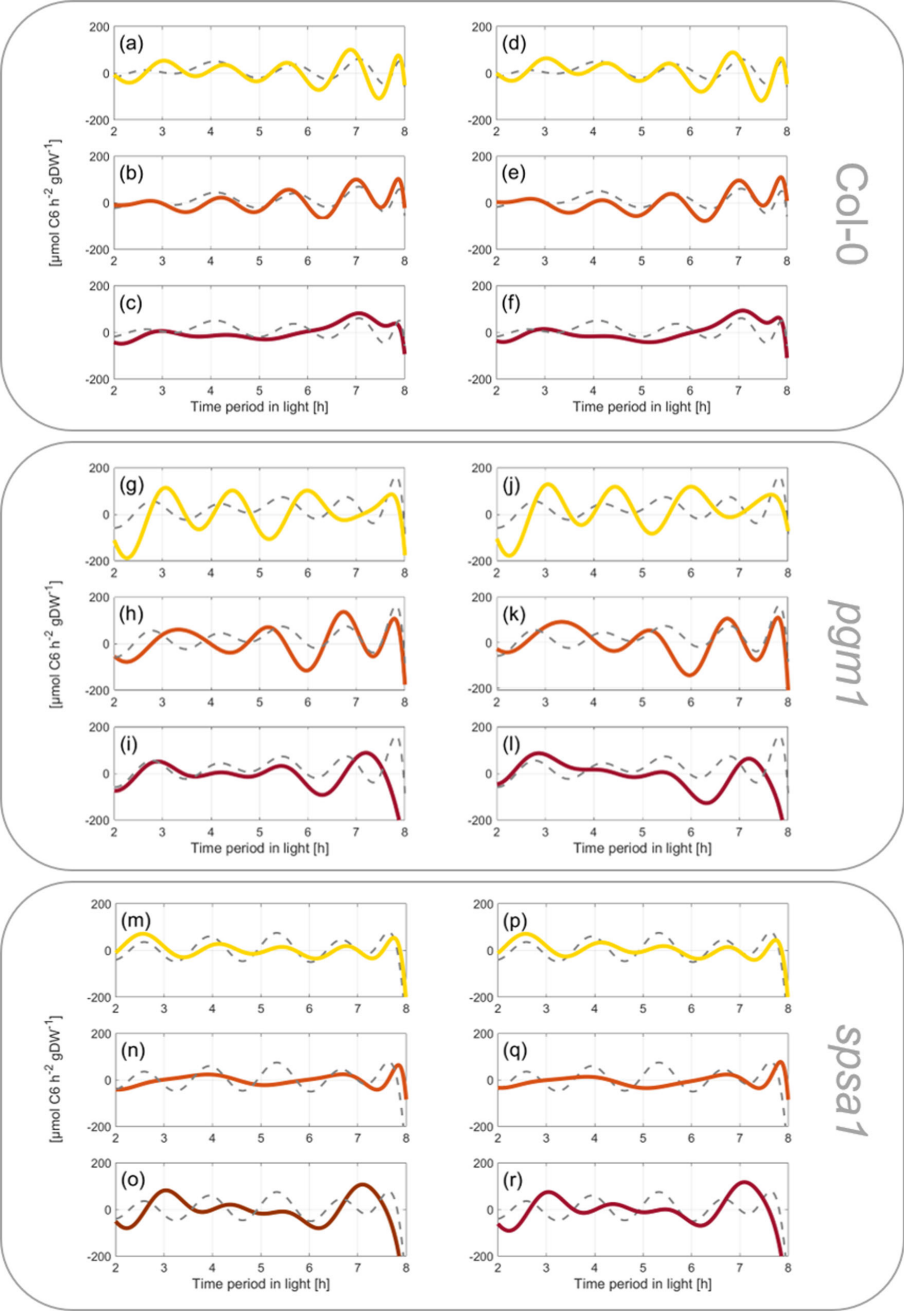
Derivatives of carbon balance equations with respect to time. Derivatives of balance equations were built for the experiments “22 °C” (control; gray dashed lines), “32 °C” (yellow lines), “36 °C” (orange lines), and “40 °C” (red lines). Upper panel: Col-0, **a**–**c** derivatives of Col-0 balance Eq. (1) (BE_1_), **d**–**f** derivatives of Col-0 balance Eq. (2) (BE_2_). In the middle: *pgm1*, **g**–**i** derivatives of *pgm1* BE_1_, **j**–**l** derivatives of *pgm1* BE_2_. Lower panel: *spsa1*, **m**–**o** derivatives of *spsa1* BE_1_, **p**–**r** derivatives of *spsa1* BE_2_.

To reveal how dynamics of carbon balance equations, which combine net photosynthesis, starch (BE_1_), and sugar metabolism (BE_2_), are affected by transient heat exposure, derivatives were built with respect to time (Fig. 8). In Col-0, increasing temperature resulted in less fluctuating derivatives of BE_1_ and BE_2_ (Fig. 8a–f). Particularly under 40 °C, oscillations were significantly damped. Also, in *pgm1*, oscillations of derivatives decreased with increasing temperature (Fig. 8g–l). Yet, particularly during the recovery phase (6 h → 8 h) from 36 °C and 40 °C to ambient temperature, fluctuations had a higher amplitude than in Col-0 (Fig. 8h, I, k, l). In *spsa1*, oscillation amplitudes were damped most notably under 36 °C. Remarkably, in all genotypes, 32 °C had the smallest observed effect on derivative oscillations compared to 22 °C (yellow lines, Fig. 8a, d, g, j, m, p).

In addition to derivative functions which revealed the absolute changing rates of balance equations, fundamental frequencies of Fourier polynomials of BE_1_ and BE_2_ further suggested a differential effect of high temperature on genotypes’ carbon balances (Supplementary Fig. S2). In Col-0, frequencies of BE_1_ and BE_2_ under 32 and 36 °C were almost doubled compared to the 22 °C experiment before they dropped in the 40 °C experiment (Supplementary Fig. S2a, b). This decrease was more emphasized in BE_2_ than in BE_1_ indicating a contribution of sugar dynamics. In *pgm1*, frequencies of BE_1_ constantly increased with temperature in experiments (Supplementary Fig. S2a). Frequencies of BE_2_ peaked under 32 °C and, finally, were lower under 40 °C than under 22 °C (Supplementary Fig. S2b). In *spsa1*, dynamics of BE_1_ and BE_2_ frequencies across experiments were similar, yet more pronounced in BE_2_. In contrast to Col-0 and *pgm1*, lowest frequency of both balance equations was observed for the 36 °C experiment.

**Fig. 9:**
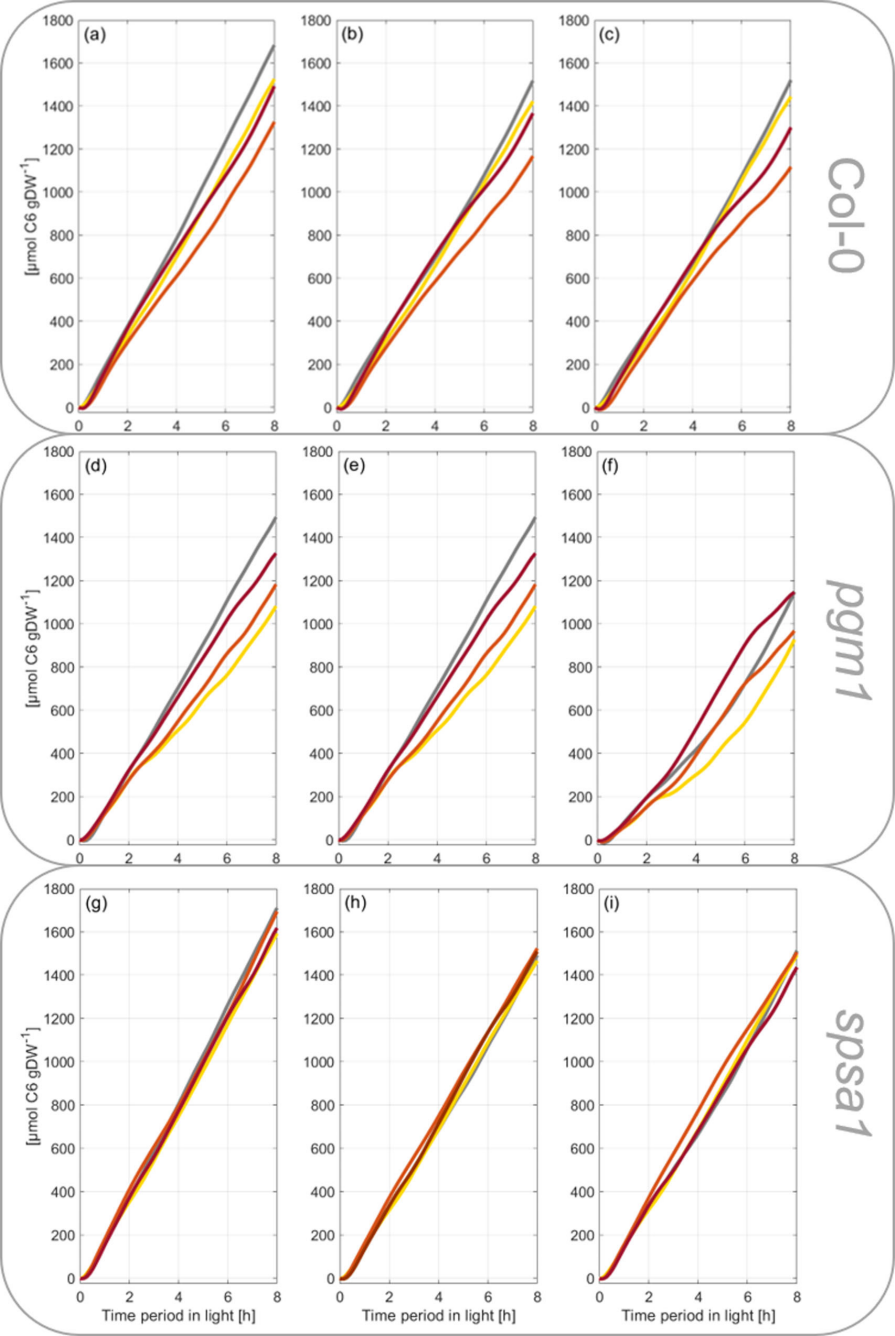
Integrals of carbon balance rates during transient heat exposure. NPS rates and rates derived from BE_1_ and BE_2_ were integrated over time to reveal the total sum of net carbon gain during the light period. **a**–**c** Col-0 integrals of NPS rates (**a**), BE_1_ (**b**), and BE_2_ (**c**). **d**–**f** *pgm1* integrals of NPS rates (**d**), BE_1_ (**e**), and BE_2_ (**f**). **g**–**i** *spsa1* integrals of NPS rates (**g**), BE_1_ (**h**), and BE_2_ (**i**). Numerical values of integrals are provided in the supplements (Supplementary Data 5).

Numerical integration of NPS rates over time period in light revealed a decreased amount of assimilated carbon due to heat exposure in Col-0 (Fig. 9a) and *pgm1* (Fig. 9d). A detailed summary of numerical values of integrals is provided in the supplements (Supplementary Data 5). In Col-0, transient heat effects on NPS rates became strongest after 2 h of temperature treatment, i.e., after 4 h in the light period. Between 4 and 6 h, particularly the amount of carbon assimilated at 36 and 40 °C deviated clearly from the 22 °C experiment (Fig. 9a). In *pgm1*, this effect was observed 2 h earlier, i.e., during the first 2 h of heat treatment between 2 h and 4 h in the light period (Fig. 9d). Surprisingly, however, the 40 °C effect on carbon assimilation was not as strong as observed for 32 and 36 °C. In *spsa1*, carbon assimilation under heat was most robust and similar to control conditions, i.e., 22 °C (Fig. 9g).

Integrals of BE_1_, which in addition to NPS rates also accounted for starch dynamics, revealed that starch dynamics in Col-0 were adjusted proportionally to affected NPS rates during transient heat exposure (Fig. 9b). In particular, integrals of 32 and 40 °C experiments became similar to the control experiment (22 °C). Due to starch deficiency, this effect was not observed in *pgm1* (Fig. 9e) while heat exposure resulted in larger integrals of BE_1_ in *spsa1* (Fig. 9h). These heat-induced effects became more pronounced in BE_2_ integrals which further accounted for net carbon flux into soluble sugar biosynthesis. In Col-0, the discrepancy of integrals between heat and control experiments was minimized during the first half of the light period, i.e., within the first 2 h of heat exposure (Fig. 9c). During the second half of the light period, discrepancy increased for 36 and 40 °C experiments. In *pgm1*, net carbon flux into sugar biosynthesis was reduced in a temperature-dependent manner which resulted in an (over-)compensation of reduced CO_2_ assimilation rates under 36 and 40 °C (Fig. 9f). Also, in *spsa1* integrals of BE_2_ increased under transient heat but this effect was less pronounced than in *pgm1* and Col-0 (Fig. 9i).

During the recovery phase, in which the temperature was set to 22 °C again (6 h → 8 h of light period), changes in starch and sugar dynamics became obvious in integrals of BE_1_ and BE_2_ for all genotypes. In Col-0, this effect was most pronounced within 36 and 40 °C experiments (Fig. 9b, c). In this phase, curves of integrals showed an inflection point directing the curve of integrals towards the control samples. In summary, this indicated reversibility of temperature-induced metabolic effects and a most robust carbon metabolism under heat in *spsa1*.

**Fig. 10:**
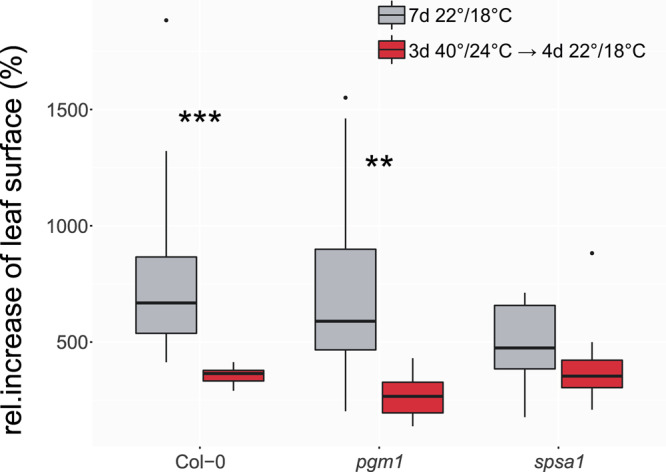
Relative increase of leaf surface during a 7-day growth period. Leaf surface was determined before and after a growth period of 7 days at 22 °C/18 °C day/night temperature (gray boxes), or after 3 days at 40 °C/24 °C followed by 4 days at 22 °C/18 °C (red boxes). Left: Col-0; middle: *pgm1*; right: *spsa1*. Box-and-whisker plots: center line, median; box limits, upper and lower quartiles; whiskers, 1.5× interquartile range; points, outliers. *n* ≥ 10. Asterisks indicate level of significance (Student’s *t* test, ***P < 0.001; **P < 0.01). Experimentally determined ratios of leaf surface are provided in the supplements (Supplementary Data 6).

To test if integrals of BE_1_ and BE_2_ can predict whole-plant performance under heat, the surface of leaf rosettes were quantified before and after heat exposure (Fig. 10; data provided in Supplementary Data 6). For this experiment, plants were grown for 5 weeks under short-day standard growth conditions (see “Methods”). Then, changes of the leaf surface of the full shoot were determined within a growth experiment in which heat exposure was prolonged to 3 days to reinforce the transient heat effect on carbon assimilation (details about the experimental design are provided in Supplementary Fig. S3). Relative increase of leaf surface of Col-0 was found to be significantly reduced by transient heat exposure (Fig. 10). Similarly, and slightly stronger, also *pgm1* was negatively affected in growth. In contrast, for plants of *spsa1* no significant heat effect on leaf surface dynamics was observed which corresponded to the observation that integrals of NPS, BE_1_, and BE_2_ were least affected by heat in *spsa1* (see Fig. 9).

## Discussion

In temperate regions, plants are frequently exposed to a changing temperature regime, and these changes might occur both over short- and long-time scales. For example, the temperature typically changes between day and night, and beyond, the temperature might also change transiently within the diurnal light and dark period. While temperature acclimation of plants typically can be observed after days of exposure to non-lethal cold or heat^16,17^, transient temperature changes and plant stress response occur within minutes or hours. Interestingly, *Arabidopsis thaliana* was found to memorize already 5 minutes of heat stress which indicates a tightly regulated molecular network involved in heat stress response^18^. High temperature, e.g., between 35 and 40 °C, is well known to result in a reduced rate of photosynthesis^19^ which has also been observed in the present study. While in Col-0 and *spsa1*, 32 °C resulted in only slightly decreased NPS rates, higher temperatures of 36 and 40 °C resulted in a significantly decreased NPS rate during the second half of the heat exposure period only in Col-0 (see Fig. 2). As previously summarized, a decreased NPS rate is not due to photosystem damage, but rather due to rubisco deactivation^19^. Consistent with this, Fv/Fm of neither genotype analyzed in the present study dropped irreversibly due to transient heat exposure (see Fig. 3). Further, consistent with previous findings which show a decreased rubisco activation at leaf temperature >35 °C^20^, the effect of 32 °C on NPS rates was much less significant than at 36 and 40 °C. In *spsa1*, however, NPS rates were less affected by heat than in Col-0 which might have several reasons. First, PSII maximum quantum yield was least affected by heat in *spsa1* (see Fig. 3). Only in *spsa1*, rates of linear electron transport (ETR), detected within a rapid light curve protocol, showed a significant increase during heat exposure suggesting a differential photosystem and/or thylakoid organization compared to Col-0 and *pgm1*. Further, compared to Col-0, *spsa1* might have had a reduced rate of photorespiration and/or mitochondrial respiration during heat exposure. While it remains speculation from our study, a higher starch accumulation rate in *spsa1* might result in a lowered respiration rate under heat because carbon equivalents may be fixed more efficiently. The observation of a destabilized NPS rate in starchless *pgm1* plants would support the stabilizing role of starch biosynthesis under transient heat exposure. Comparison of transpiration revealed similar rates across all genotypes, which suggests that observed NPS effects are unlikely due to differential stomata closure and/or secondary effects like leaf cooling^21,22^. However, previous reports under ambient conditions have shown that SPS knockout mutants have rather enhanced than lowered dark respiration rates which do not directly support the hypothesis of NPS stabilization by starch biosynthesis^23^. Another explanation might be a secondary effect of the *spsa1* mutation on rubisco and/or rubisco activase which, to our knowledge, has not been shown in current literature but which needs to be proven in future studies.

While sucrose and glucose metabolism showed a dynamic and differential accumulation profile between 32, 36, and 40 °C experiments, dynamics of fructose concentrations were most conserved across all temperature treatments and, remarkably, also across genotypes (see Fig. 7). An initial accumulation within the first 2 h of the light period was followed by a significant decrease until the end of heat exposure and an accumulation during the recovery phase between 6 and 8 h of the light period. Only *pgm1* showed a differential pattern at 22 and 32 °C but became similar in its fructose profile to Col-0 and *spsa1* under 36 and 40 °C. In mature *Arabidopsis* leaves, fructose levels are significantly affected by invertases that catalyze the hydrolysis of sucrose and release free hexoses^24^, and by fructokinase catalyzing ATP-dependent phosphorylation which yields fructose-6-phosphate^25^. As fructose and glucose profiles differed in the present study, this cannot (solely) be explained by invertase reactions which release equimolar concentrations of both hexoses. However, differential regulation of hexokinase and fructokinase could explain the different hexose profiles. Fructokinase yields the direct substrate for glycolysis, TCA cycle, and mitochondrial respiration. In a previous study that analyzed transcript levels in *Arabidopsis thaliana* under combined drought and heat stress found increased transcripts for both hexokinase and fructokinase^26^. Although the experimental design differed significantly from this study, together with other findings this suggests a central role of hexose phosphorylation in heat stress response and acclimation^17^. As leaf respiration rates typically increase under elevated temperature^27^, observed consistent fructose dynamics might be due to a relatively high rate of glycolytic consumption under transient heat exposure.

Integrating net CO_2_ assimilation rates with starch and sugar turnover allows for balancing of the central carbohydrate metabolism. In this context, Fourier polynomials support the functional and time-continuous estimation of dynamics of metabolism. Integration and differentiation of Fourier polynomials is straightforward, and, at the same time, provides a comprehensive mathematical framework that is applied in diverse fields of natural sciences and engineering^28–30^. As described in the block diagram model (see Fig. 1), metabolic dynamics were simulated by the addition of Fourier polynomials comprising input functions (NPS rates) and consuming functions (starch and sugar dynamics). With such a design, dynamics of plant carbon balancing become traceable without the need for the application of composed spline functions. Further, the properties of Fourier polynomials can reveal further insight into metabolic regulation and consequences of environmental changes. For example, in the present study both amplitude and frequency of derivatives of balancing equations differed with regard to genotype and environment. A different pattern was observed in Col-0 and *spsa1* than in *pgm1*, indicating that the starchless mutant has a less buffered metabolic response towards heat stress than both other genotypes. This was supported by the comparison of fundamental frequencies of BE_1_ and BE_2_ Fourier polynomials. Here, a genotype-specific pattern was observed which reflected the impact of starch deficiency in *pgm1* and comparatively high metabolic dynamics in *spsa1* within the 40 °C experiment (see Fig. 8 and Supplementary Fig. S1). Thus, summarizing the effects of transient heat on NPS rates and carbohydrate metabolism resulted in characteristic Fourier polynomials which enabled the discrimination of genotypes by their derivatives and fundamental frequencies. Genotypes could further be discriminated by integrals of Fourier polynomials derived from very short (4 h), transient temperature profiles. A prolonged heat exposure over 8 h and 3 days (with decreased night temperature) finally resulted in measurable and significant differences in leaf size as a proxy for plant growth. Hence, although the experimental design of heat treatment was changed in the growth experiments (compare Supplementary Figs. S3 and S5), this emphasizes the suitability of such a modeling approach to detect and quantify (relatively) small differences in balance equations over short time periods (see Fig. 9), and to predict significant effects of dynamic plant-environment interactions on (long-term) plant performance and physiology (see Fig. 10).

Fourier analysis and spectra of frequencies have been applied before in a different context, e.g., to analyze gene-expression time-series data^31^. These authors coupled Fourier analysis to supervised learning algorithms to discriminate between housekeeping genes and non-housekeeping genes in HeLa cells. This example provides evidence for the suitability of Fourier analysis to be combined with machine learning algorithms which is of particular interest for large-scale data sets. However, also data sets with only a relatively low number of variables may need mathematical functions for quantitative analysis and integration, e.g., as shown in the present study. This is due to the need for combining dynamics of variables rather than steady-state values under one condition. Here, Col-0, *pgm1,* and *spsa1* could successfully be discriminated by the dynamics of fundamental frequencies of Fourier polynomials across different experiments rather than by one absolute value of a frequency. This observation further emphasizes the need for a functional mathematical description of experimentally observed system dynamics because underlying attributes, e.g., monotonicity or curvature, can be derived from such a description. These attributes provide valuable information about system properties like stability or predictability which need to be essentially addressed for predictive modeling^32,33^. Conclusively, Fourier polynomial-based balance modeling provides a mathematical approach that can essentially support nonlinear modeling of metabolism, and which might, in future studies, even serve as a mathematical framework to connect oscillations in metabolism with quantum theory^34,35^.

## Methods

### Plant cultivation and stress treatment

Plants of *Arabidopsis thaliana*, accession Columbia-0 (Col-0), *spsa1*(AT5G20280, SALK line 148643C) and *pgm1* (AT5G51820; TAIR stock CS3092) were grown on a 1:1 mixture of GS90 soil and vermiculite in a climate chamber under short-day conditions (8 h/16 h light/dark; 100 µmol m^−2^ s^−1^; 22 °C/18 °C; 60% relative humidity). The *spsa1* line was confirmed via PCR to be homozygous and activity was found to be decreased to 30-50% of the wildtype Col-0 (Supplementary Fig. S4). The *pgm1* mutant had a dwarf phenotype and starch content was below the detection limit. After 4 weeks, plants were transferred to a growth cabinet (Conviron^®^, www.conviron.com) and grown for 2 further weeks under short-day conditions with the same settings as in the climate chamber. After 6 weeks, on the day of sampling, the temperature in the growth cabinet was kept at 22 °C during the first 2 h in the light (0 h → 2 h, 22 °C). Then, in three independent experiments, the temperature was increased to (i) 32 °C, (ii) 36 °C, or (iii) 40 °C for a total of 4 h (2 h → 6 h, temperature increase). In the control experiment, the temperature was set constantly to 22 °C. Between 6 and 8 h, i.e., until the end of the light period, the temperature was set to 22 °C in all experiments. A graphical representation of the experimental setup is provided in Supplementary Fig. S5. Plants were sampled at each time point (0 h, 2 h, 6 h, 8 h) by cutting the full leaf rosette at the hypocotyl. Samples were immediately frozen in liquid nitrogen and stored at −80 °C until further use.

### Pulse-amplitude modulation and quantification of net CO_2_ uptake

Maximum quantum yield of photosystem II (Fv/Fm) and electron transport rates (ETR) were quantified by pulse-amplitude modulation (PAM) using a WALZ^®^ Junior-PAM (Heinz Walz GmbH, Effeltrich, Germany, https://www.walz.com/). Plants were dark incubated at 22 °C for 15 min prior to measurements. After dark incubation, Fv/Fm was determined by applying a saturating light pulse (photosynthetic photon flux density (PPFD) = 4000 µmol m^−2^ s^−1^). A rapid light curve protocol was applied to quantify ETR, qP and qN under increasing PPFD^36^. Sequentially, every 20 s, actinic irradiance was increased from 0 up to 2250 µmol photons m^−2^ s^−1^ (0, 38, 68, 98, 135, 188, 285, 428, 630, 938, 1230, 1725, 2250 µmol photons m^−2^ s^−1^).

Rates of net photosynthesis were recorded within the Conviron^®^ growth cabinet using a WALZ^®^ GFS-3000FL system equipped with measurement head 3010-S (Heinz Walz GmbH, Effeltrich, Germany, https://www.walz.com/). Temperature, light, and humidity control of the measurement head were set to follow ambient conditions, i.e., to follow surrounding growth cabinet conditions. A summary of recorded temperature, light, and humidity curves is provided in the supplement (Supplementary Fig. S6). Rates of transpiration were recorded together with net CO_2_ uptake and are summarized in the supplement (Supplementary Fig. S1). For each genotype and growth condition, i.e., temperature setup, three independent samples were measured.

### Extraction and quantification of carbohydrates

Plant material was ground to a fine powder under constant freezing with liquid nitrogen. The powder was lyophilized for three days and subsequently used for carbohydrate analytics. Starch and soluble carbohydrates were extracted and photometrically determined as described before^37^. Plant powder was incubated with 80% ethanol at 80 °C for 30 min. After centrifugation, the supernatant was transferred to a new tube, and extraction was repeated with the pellet. Supernatants were unified and dried in a desiccator. The starch-containing pellet was hydrolyzed with 0.5 M NaOH for 45 min at 95 °C. After acclimation to room temperature, 1 M CH_3_COOH was added and the suspension was digested with amyloglucosidase solution, finally releasing glucose moieties from starch granules. Glucose was photometrically determined by applying a coupled glucose oxidase/peroxidase/o-dianisidine assay.

Soluble sugars sucrose, glucose, and fructose were determined from dried ethanol extracts after dissolving in water. After incubation with 30% KOH at 95 °C, sucrose was quantified using an anthrone assay. Anthrone was dissolved in 14.6 M H_2_SO_4_ (0.14% w/v), incubated with the prepared sample for 30 min at 40 °C and absorbance was determined photometrically at 620 nm. Glucose amount was determined photometrically by a coupled hexokinase/glucose 6-phosphate dehydrogenase assay resulting in NADPH + H^+^ at 340 nm. For fructose quantification, phosphoglucoisomerase was added to the reaction mixture after glucose determination.

### Quantification of SPS activity

The activity of sucrose phosphate synthase (SPS) was determined using the anthrone assay^37^. In brief, freeze-dried leaf tissue was suspended in extraction buffer containing 50 mM HEPES–KOH (pH 7.5), 10 mM MgCl_2_, 1 mM EDTA, 2.5 mM DTT, 10% (v/v) glycerol and 0.1% (v/v) Triton-X-100. Following incubation on ice, extracts were incubated for 30 min at 25 °C with a reaction buffer containing 50 mM HEPES–KOH (pH 7.5), 15 mM MgCl_2_, 2.5 mM DTT, 35 mM UDP-glucose, 35 mM F6P, and 140 mM G6P. Reactions were stopped by adding 30% KOH and heating to 95 °C. Sucrose was determined photometrically after incubation with anthrone in H_2_SO_4_.

### Statistics and reproducibility

Statistical analysis was performed in R and R Studio (www.r-project.org)^38^. Fourier series fitting was done within MATLAB^®^ (www.themathworks.com), and block diagram models were created in Simulink^®^ (www.themathworks.com). Plant total leaf surface was quantified using the Fiji software)^39^ with the SIOX plugin (https://imagej.net/plugins/siox). The sample size was chosen according to (maximal) measurement and growth capacities (most limiting: growth cabinet, gas analyzer). Replicates represent biological replicates which were treated independently from each other to test and validate reproducibility. Plants were grown in pots with randomized order within the climate chamber to minimize or exclude any position or sampling effect. Samples were randomly chosen for molecular analysis. Investigators were blinded to group allocation during data collection.

### Reporting summary

Further information on research design is available in the Nature Research Reporting Summary linked to this article.

## Supplementary information

Supplementary Information

Description of Additional Supplementary Files

Supplementary Data 1

Supplementary Data 2

Supplementary Data 3

Supplementary Data 4

Supplementary Data 5

Supplementary Data 6

Reporting Summary

## Data Availability

Data presented in this study are provided within Supplementary Data files 1–6.
