# Supplementary material for: Predicting plant growth response under fluctuating temperature by carbon balance modelling
Source: Commun Biol. 2022 Feb 24;5:164. doi: 10.1038/s42003-022-03100-w (PMC8873469; doi:10.1038/s42003-022-03100-w)
Supplement: Supplementary file 1 — Supplementary Information [file 42003_2022_3100_MOESM1_ESM.pdf]

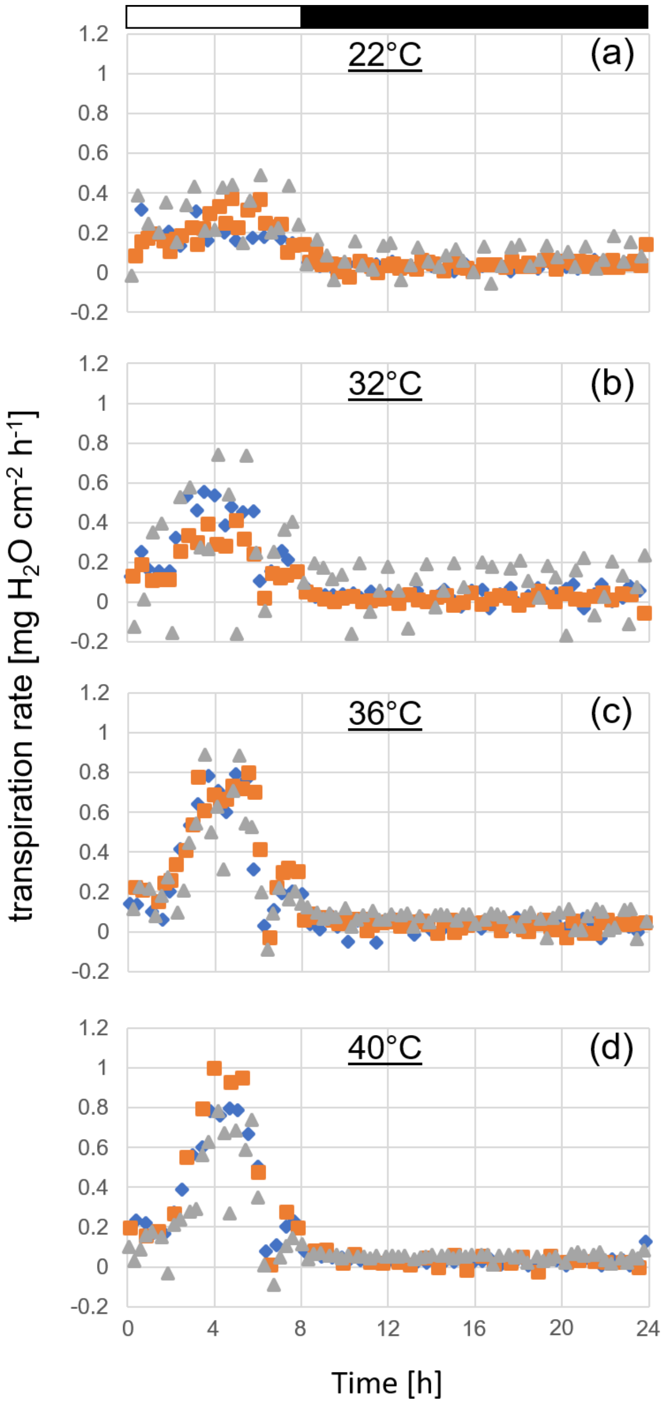

Figure S1. Mean transpiration rates recorded under each condition (n=3). (a) Transpiration rates at 22°C; (b) transpiration rates at 32°C; (c) transpiration rates at 36°C; (d) transpiration rates at 40°C. Blue diamonds: Col-0; orange squares: spsa1; grey triangles: pgm1. White and black bars on top indicate light and dark phase (8h/16h).

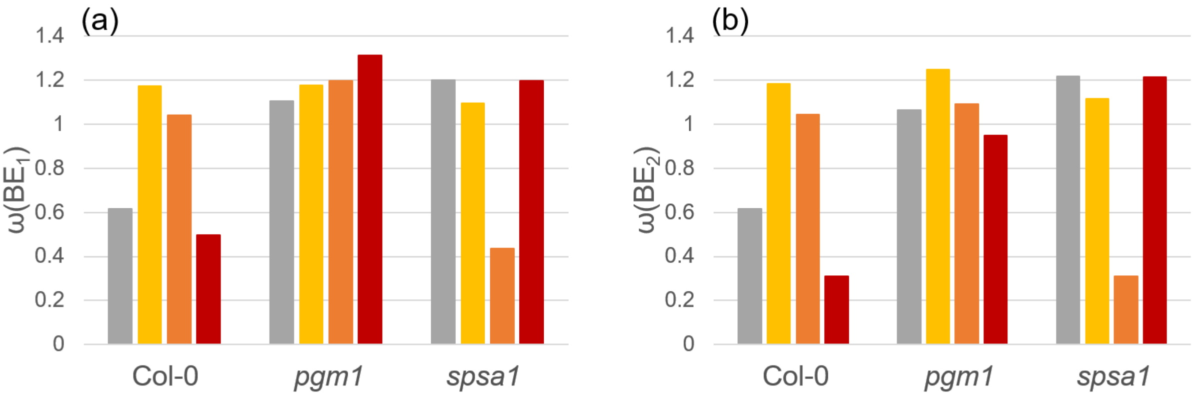

Figure S2. Fundamental frequencies of Fourier polynomials of BE1 (a) and BE2 (b). Frequencies were calculated as the ratio of (2π/T) where T represents periods. Bar colour indicates different experiments. Grey bars: 22°C experiment; yellow bars: 32°C experiment; orange bars: 36°C; red bars: 40°C experiment.

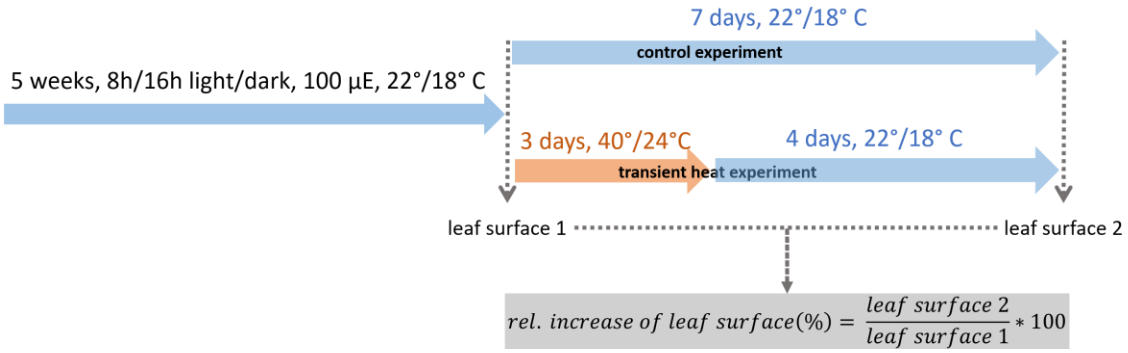

Figure S3. Design of growth experiment for leaf surface quantification under prolonged transient heat.

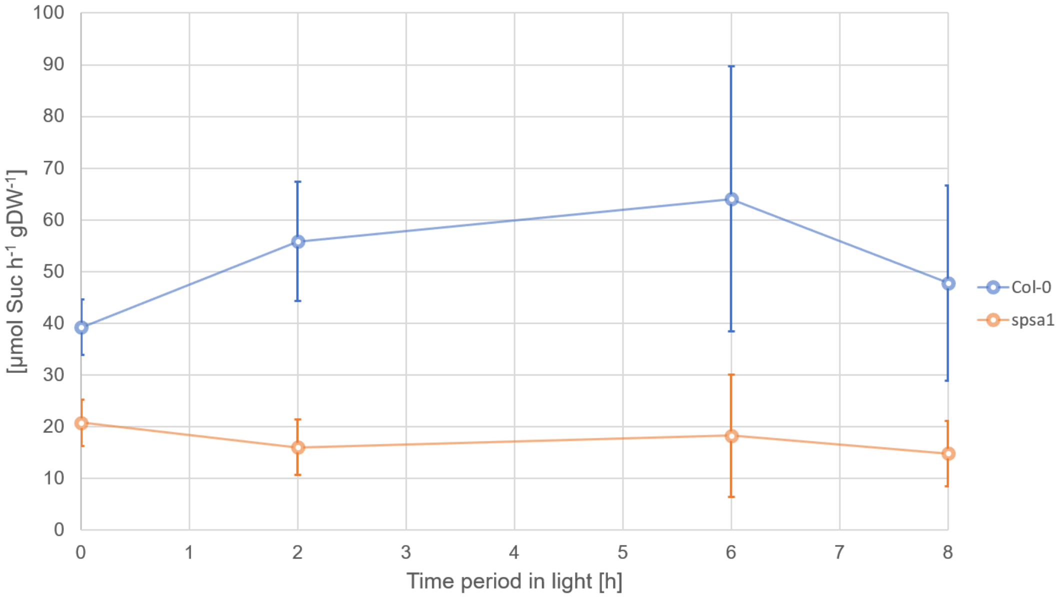

Figure S4. SPS maximum activity under substrate saturation in Col-0 and spsa1. Error bars represent means +/- SD (n=5).

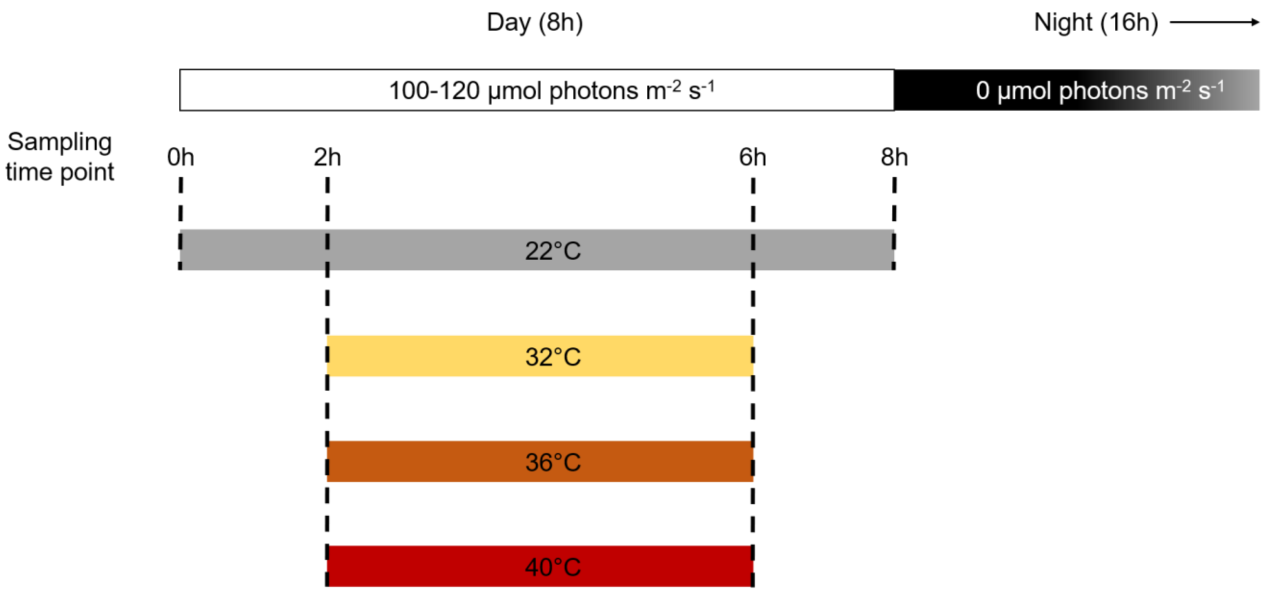

Figure S5. Schematic representation of the experimental design used in this study.

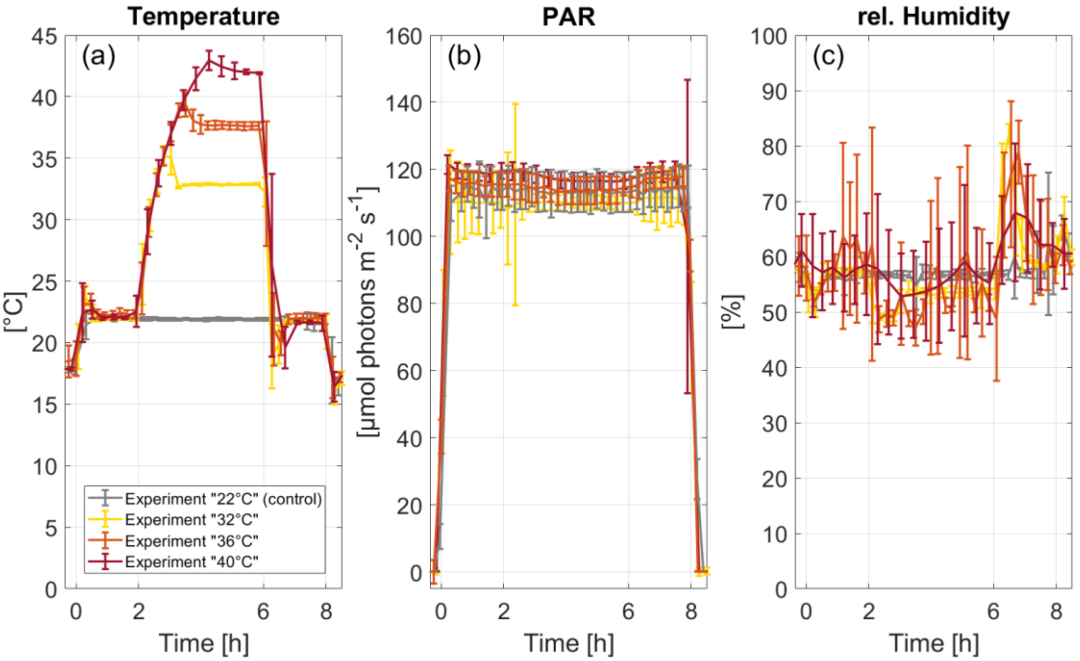

Figure S6. Summary of actual growth cabinet parameters during sampling period. (a) Temperature, (b) PAR, (c) relative humidity. Error bars represent means +/- SD (n = 3).
