# Supplementary material for: Predicting plant growth response under fluctuating temperature by carbon balance modelling
Source: Commun Biol. 2022 Feb 24;5:164. doi: 10.1038/s42003-022-03100-w (PMC8873469; doi:10.1038/s42003-022-03100-w)
Supplement: Supplementary file 2 — Description of Additional Supplementary Files [file 42003_2022_3100_MOESM2_ESM.pdf]

Description of Additional Supplementary Files

File name: Supplementary Data 1

Description: Fourier polynomials and balance equations.

File name: Supplementary Data 2

Description: Source data for Figure 2.

File name: Supplementary Data 3

Description: Source data for Figure 3 and Figure 4.

File name: Supplementary Data 4

Description: Metabolite data for Figure 5.

File name: Supplementary Data 5

Description: Results of numerical integration of net carbon assimilation related to Figure 9.

File name: Supplementary Data 6

Description: Source data for Figure 10.
